# Extraction of business relationships in supply networks using statistical learning theory

**DOI:** 10.1016/j.heliyon.2016.e00123

**Published:** 2016-06-21

**Authors:** Yi Zuo, Yuya Kajikawa, Junichiro Mori

**Affiliations:** a Institutes of Innovation for Future Society, Nagoya University, Nagoya, Aichi, Japan; b School of Environment and Society, Tokyo Institute of Technology, Tokyo, Japan; c Policy Alternatives Research Institute, The University of Tokyo, Tokyo, Japan

**Keywords:** Computer science, Social sciences, Applied sciences

## Abstract

Supply chain management represents one of the most important scientific streams of operations research. The supply of energy, materials, products, and services involves millions of transactions conducted among national and local business enterprises. To deliver efficient and effective support for supply chain design and management, structural analyses and predictive models of customer–supplier relationships are expected to clarify current enterprise business conditions and to help enterprises identify innovative business partners for future success. This article presents the outcomes of a recent structural investigation concerning a supply network in the central area of Japan. We investigated the effectiveness of statistical learning theory to express the individual differences of a supply chain of enterprises within a certain business community using social network analysis. In the experiments, we employ support vector machine to train a customer–supplier relationship model on one of the main communities extracted from a supply network in the central area of Japan. The prediction results reveal an F-value of approximately 70% when the model is built by using network-based features, and an F-value of approximately 77% when the model is built by using attribute-based features. When we build the model based on both, F-values are improved to approximately 82%. The results of this research can help to dispel the implicit design space concerning customer–supplier relationships, which can be explored and refined from detailed topological information provided by network structures rather than from traditional and attribute-related enterprise profiles. We also investigate and discuss differences in the predictive accuracy of the model for different sizes of enterprises and types of business communities.

## 1 Introduction

Customer–supplier relationships are conventionally modeled and analyzed based on a linear structure (Handfield and Nichols, 1999) or dyadic structure (Cox et al., 2001) in the field of supply chain management. As goods and materials are vertically delivered between enterprises, it appears to be straightforward to model supply chains through the extrapolation of linear perspectives on customer–supplier relationships. However, oversimplifying the linear concept of the supply chain poorly reflects the complex and cyclical structure of customer–supplier relationships, and the linear model is also inappropriate for managers and academics to use to analyze and design supply chain developments (Kim et al., 2011). Therefore, both managers and academics strive to develop management strategies to improve the supply chain and to lead enterprises not only towards stability and profitability, but also towards the adoption of sustainable and innovative business partners (Zhu and Sarkis, 2004, Bellamy et al., 2014). Recently, several studies have attempted to improve this linear structure by using the supply network as an alternative approach (Turnbull et al., 1996, Shrivastava, 1995), and growing interest in applications of social network analysis (SNA) to the supply chain has rendered it a preferable model to linear analysis (Autry and Griffis, 2008, Borgatti and Li, 2009, Carter et al., 2007). Nevertheless, due to real supply network data, the development of the network concept and the validation of its availability in practice have not been explicitly considered. On the other hand, applications of SNA for business developments are needed by enterprises to provide efficient and effective support for the identification of latent business partners with respect to current business contexts and future business trends.

To address these limitations, we propose an SNA-based model for predicting customer–supplier relationships for a real supply network in Japan (see the Tokyo Shoko Research Limited website http://www.tsr-net.co.jp/en for more information on the data). Based on previous studies (Chou and Chang, 2008, Hu and Zhang, 2008, Mori et al., 2012), this article also employs statistical learning theory and a support vector machine (SVM) (Vapnik, 1995) to predict customer–supplier relationships. In comparison with these studies, among which Mori et al. (2012) designed and trained an SVM model using linear kernel accounting where the size of features was much greater than the size of training instances, several nonlinear kernel tricks are examined and compared with respect to the predictive accuracy level, as we only design dozens of features. Since the kernel of the Gaussian radial basis function (RBF) can transform an original finite-dimensional space into an infinite-dimensional space, the highest predictive accuracy can be obtained by using an RBF kernel with σ=1.0, which can generate an insightful classifier hyperplane relative to other kernels in order to rationalize consumer–supplier relationship extraction. We then introduce the network centrality concept and develop the SVM model using network centralities (NCs). NCs are used to determine the importance and influence of each node in an embedded network structure, to identify a new alternative customer–supplier relationship (related enterprise attributes (EAs) are introduced as explanatory variables in Mori et al. (2012)'s study). When we introduce closeness and betweenness into the SVM model to predict consumer–supplier relationships, the predictive accuracy of our model, which combines EAs with NCs, is significantly improved by 5.63% (from 76.41% to 80.71%). On the other hand, we also find that network centralities can dramatically improve the predictive accuracy levels, when enterprises are separated and grouped using an optimal algorithm of community division. We employ a fast modularity maximization algorithm, the Newman method (Girvan and Newman, 2002, Clauset et al., 2004), to detect sub-communities in the main supply network community, and four sub-communities are detected from the original community. After introducing the NCs with EAs, the predictive accuracy of the original community is improved by 5.63%, and the predictive accuracy level of each sub-community are improved by 7.18%, 9.01%, 8.15% and 9.39%, respectively. Furthermore, we estimate the network centrality degree as the separating indicator in place of the capital or employee number, to separate different enterprises into two classes; namely large enterprises (LEs) and small & medium enterprises (SMEs). As the degree is defined as the number of edges through which a node connects to other nodes in the network, enterprises that sustain more relationships (with customers or suppliers) are more active and central in the supply network. Our experimental results show that the predictive accuracy of both LEs and SMEs can be dramatically improved by combining NCs with EAs. More especially, the results obtained for the SMEs when only using NCs alone are sufficiently accurate.

This article makes two contributions. It first applies a machine learning approach using EAs and NCs as inputs thus introducing a new methodological approach to the literature on supply chain design and management. We also found that applying a combination of these two types of features is effective for predicting customer–supplier relationships, demonstrating the effectiveness of our proposed methodology, which has not been reported so far. Second, we analyze the effectiveness of our methodology when applied to LEs and SMEs. As considering EAs alone, prediction performance levels are high when LEs are customers and SMEs are suppliers. This is typically the case of supply chains. However, it is difficult to make reliable predictions for the other cases. In this article, we show that integrating NCs with EAs facilitates the prediction of customer–supplier relationships as SME–LE and SME–SME. Our findings, we believe, can contribute to the design of supply chains and can provide insight on ways of further developing business partner recommendation systems via machine learning.

The remainder of the article is organized as follows. We present a review of related literature on previous studies of SNA applied to supply networks in Section 2. In this section, we also discuss recent research topics on business data mining. The methodology employed in this article is explained in Section 3. Section 4 presents the experiments and a discussion, and Section 5 provides conclusions and a summary of the results.

## 2 Background

### 2.1 Empirical approaches to SNA applied to supply chains

From a network perspective, supply chains do not represent a focal enterprise's direct link to each of its business partners (e.g., suppliers and customers) but its indirect or circular links to invisible enterprises that are associated with its business partners. This view of the supply chain is relatively new to business management theory and its effectiveness has been proven through both anecdotal and theoretical evidence (e.g., Japanese manufacturers and assemblers) (Cox et al., 2001, Kajikawa et al., 2010). Research on supply networks has revealed the proactive and collaborative facets of supply network management (Bellamy et al., 2014).

In this supply network structure, the relative positioning of each enterprise with respect to others depends on the extent to which an enterprise affects both strategies and behaviors. In this context, the supply network is crucial for analyzing each enterprise's role and importance based on its embedded position in the broader relationship structure (Kim et al., 2011).

Currently, SNA has increasingly gained acceptance by both scholars and managers for its potential merits in integrating the operations and supply management fields. According to Cox et al. (2001), SNA is a collaborative and developmental approach to the integration of supply chain management that can reduce transactional path length and that is more focused on the eradication of waste and supply chain inefficiency. The SNA concept is also particularly suited to studying how customer–supplier relationships in a supply network account for competitive advantages through the management of materials movement and information diffusion (Borgatti and Li, 2009).

Until recently, SNA has not been explicitly applied in an empirical study of real supply networks, and there is a general paucity of SNA applications in supply management with only a few exceptions of a small corpus (Kim et al., 2011). In particular, no existing works apply major network centralities within a machine learning approach, which can offer practical applications and quantitative analyses of supply networks when identifying potential business partners. Therefore, in this article, we replace the traditional linear supply chain with an alternative supply network at the node- and network-levels, and we introduce machine learning techniques to illustrate the specific roles played by network centralities in a supply network.

### 2.2 Recent research on business data mining

Business plan development and strategy principles are perdurable research subjects in the fields of economics, finance, and management. Predictions based on data mining and machine learning technologies have been a primary focus of these fields for over half a century. Artificial neural networks (ANNs) are some of the most widely used models for predicting stock prices, bankruptcy trends, etc., in these fields (Atiya, 2001, Enke and Thawornwong, 2005). In addressing such real-world problems, probabilistic models such as the hidden Markov models (HMMs) (Hassan, 2009) and Bayesian networks (BNs) (Sarkar and Sriram, 2001, Zuo and Kita, 2012) are widely used not only for prediction, but also as expert systems or decision support systems for academic R&D. Since the 2000s, as support vector machines (SVMs) have been increasingly recognized for their key role in machine learning, more and more applications have been proposed in the fields of management and marketing (Hu and Zhang, 2008, Guo et al., 2009, Chen and Fan, 2012).

More recently, the application of machine learning technologies to contexts of supply chain management has been investigated. For example, Carbonneau et al. (2007) investigate the applicability of advanced machine learning techniques (e.g., ANN and SVM) to forecast distorted demand at the end of a supply chain; Chou and Chang (2008) proposed a decision support system based on a strategy-aligned fuzzy approach for solving supplier/vendor selection problems from perspectives of strategic supply chain management; and other authors used an SVM to forecast customer–supplier relationships (Guo et al., 2009, Hu and Zhang, 2008, Mori et al., 2012). However, real world data are difficult to obtain before actual transactions are contracted and launched (Mori et al., 2012). In obtaining actual data on each enterprises from commercial business databases, Mori et al. (2012) designed features of customer–supplier relationships and proposed machine learning instances via a web system that can automatically recommend a list of potential business partners for a given enterprise.

Among these previous studies, Mori et al. (2012) utilized an integrated SVM for customer–supplier relationships to predict focal relationships. Fewer studies utilize the relational features of business data mining; however, Mori et al. (2012)'s research is closely related to the link-prediction problem. For link-prediction problems, the utilization of relational features intrinsic to a network can draw meaningful inferences from observed network data. Therefore, this article proposes an SNA-based prediction model of customer–supplier relationships. We apply a machine learning approach that captures the effects of both EAs and NCs, and we familiarize the readers with this new methodological approach as a contribution to the literature on supply chain design and management.

## 3 Methodology

### 3.1 Basic concept

**Figure 1:**
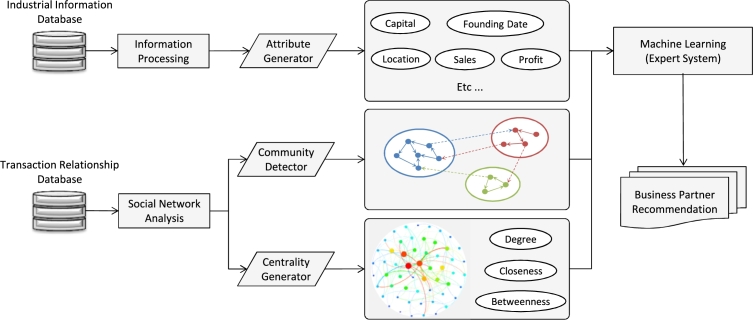
Overview of the SNA-based prediction proposal for finding business partners.

Figure 1 presents the basic principles of our methods, which involve applying SNA techniques to supply network analysis. Two databases are used as raw data. One records industrial information on enterprises, and the other records transaction relationships among them. Both are associated with preprocessing and are transferred to attribute generator, community detector and centrality generator.

Industrial data are managed during the information processing and are then transformed into enterprise attributes (e.g., capital, founding date, etc.) by using an attribute generator. Transaction relationships are managed through the SNA processing, and are then separated over two phased of sub-processing. During the first round of sub-processing, supply networks based on transaction relationships are transferred to a community detector, where obtained communities are densely connected inside but are sparsely connected to each other communities outside. During the second round of sub-processing, the supply network is transferred to a centrality generator, where network centralities (e.g., degree, closeness and betweenness) are determined for each enterprise. In the supply network, enterprises and their transaction relationships are represented by nodes and links, respectively, and centralities reveal their features and positions in the network as shown in Figure 1. (In the network obtained from the centrality generator, nodes are colored and sized by degree.)

Finally, we propose a prediction model based on enterprise attributes, communities and network centralities using machine learning techniques. During this processing, we estimate the parameters of the model. Recommendation, prediction and estimation results for identifying business partners are extracted.

**Figure 2:**
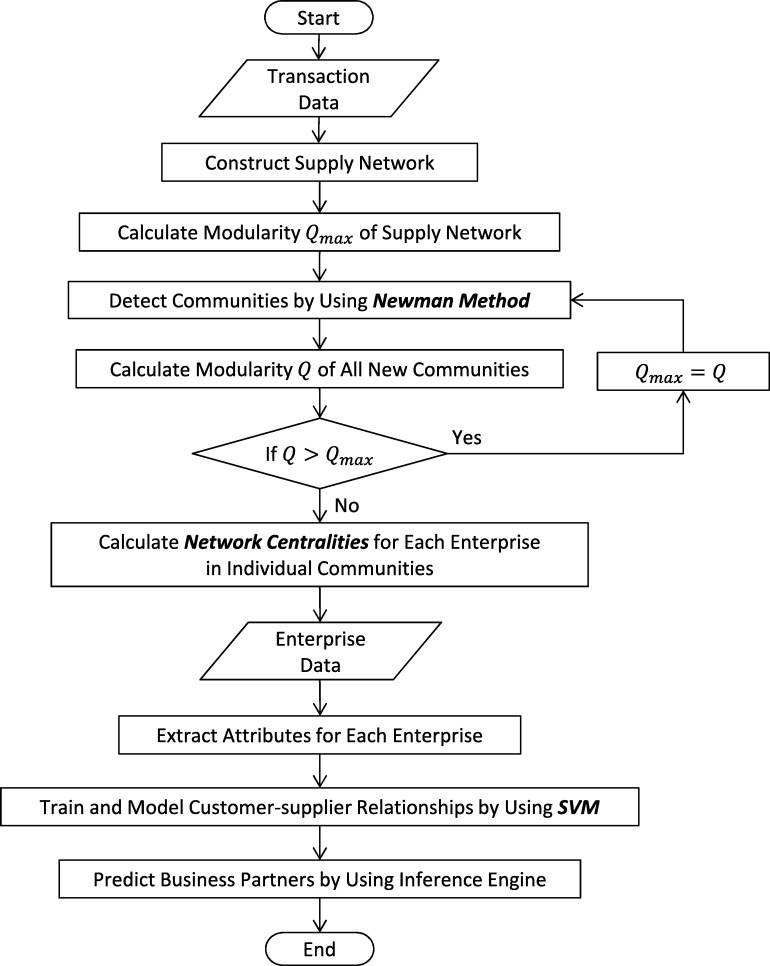
Flowchart of the proposed algorithm.

The algorithm of our proposal is illustrated in Figure 2 and is summarized as follows:

1. Input the transaction data and construct a supply network based on these data.
2. Calculate modularity levels of the current supply network and save them as $Q_{\max}$.
  (a) Use the Newman method (Clauset et al., 2004) to detect and separate communities in the supply network.
  (b) Calculate modularity levels for all new communities and save them as *Q*.
  (c) If $Q>Q_{\max}$, $Q_{\max}=Q$ and one may progress to step (a).
3. Calculate network centralities for each enterprise in individual communities.
4. Input the enterprise data and extract attributes for each enterprise from these data.
5. Use an SVM to train and model customer–supplier relationships based on transaction and enterprise data.
6. Use this inference engine to predict and find latent business partners.

### 3.2 Newman method

Real world networks such as food web, social media, and citations often have community structures. Nodes represent entities (e.g., animals, persons, and articles), and edges represent interactions (e.g., feeding, communicating, and citing); Therefore, entities are characterized by node connections of the same community with dense edges compared to nodes in other communities connected by sparse edges. Clauset et al. (2004) propose a fast algorithm, the Newman method, that involves employing a greedy search without repeating calculations for each edge. The Newman method is a fast modularity algorithm for community structure inference that maximizes modularity *Q* as follows:

(1) $$Q=\sum_{i}(e_{ii}-{\left(\sum_{j}e_{ij}\right)}^{2})=\sum_{i}(e_{ii}-a_{i}^{2}),$$

where $e_{ii}$ denotes the fraction of edges in community *i*, $e_{ij}$ denotes the fraction of edges that link nodes in community *i* to nodes in community *j*, and $a_{i}$ denotes the fraction of edges that link nodes in community *i* to all other communities.

The operation process of the Newman method (Clauset et al., 2004) is a hierarchical agglomeration algorithm that detects community structures and that focuses on changes Δ*Q* in modularity as

(2) $$\Delta Q=e_{ij}+e_{ji}-2a_{i}a_{j},$$

which is calculated for either pair of communities in a network and selects the largest Δ*Q* of those that combine them. The Newman method proposed by Clauset et al. (2004) can be described as follows:

1. Calculate modularity as *Q* for the initial network (all nodes are independent).
2. Calculate Δ*Q* for pairs of communities (or nodes).
3. Select the largest Δ*Q* to combine, and add it to *Q*.
4. Repeat steps 2 to 4 until *Q* has no more increments.

### 3.3 Network centrality

According to the social network perspective, a network consists of entities (i.e., enterprises) represented by nodes, and the ties (i.e., customer–supplier relationships) that link them represented by links. In a supply network context, entities reflect customers and suppliers linked by activities related to the procurement and transformation of raw materials for producing and delivering goods and services.

#### 3.3.1 Degree centrality

The degree is the simplest centrality measure of network theory and is defined as the number of links incident upon a node:

(3) $$C_{D}(v_{i})=\sum_{j=1,i\neq j}^{n}\wedge (v_{i},v_{j})$$

where for a given node $v_{i}$, ∧(⋅)=1 when a link exists between $v_{i}$ and $v_{j}$. Otherwise, ∧(⋅)=0. Although degree centrality can be simply calculated using Eq. (3), it is intuitive and interpretable to measure the importance (e.g., activity and cohesiveness) of a node. Particularly for a directed network, the degree is separated into indegree and outdegree measures, which denotes the number of links to a node from others and the number of links to other nodes from that node, respectively. In a supply network, an enterprise with a higher degree centrality value is recognized as an enterprise with more direct contacts than other enterprises and thus has the potential to affect others through operational decisions and strategic behavior (Kim et al., 2011).

#### 3.3.2 Closeness centrality

Closeness was first proposed by Sabidussi (1966), and is conceptually the simplest measure that identifies the centrality of a point by summing the geodesic distances from a point to all other points in a network. For a given point $v_{i}$ closeness is given by

(4) $$C_{C}(v_{i})=\frac{1}{\sum_{j=1,i\neq j}^{n}d(v_{i},v_{j})}$$

where $d(v_{i},v_{j})$ denotes the number of edges in the shortest path linking $v_{i}$ to $v_{j}$, and when finding the shortest path, all links are considered to be undirected. In contrast to degree, which presents the egocentric centrality measures of a node, closeness centrality is a sociocentric measure of a node rather than an egocentric measure (Marsden, 2002). Eq. (4) shows that node $v_{i}$ with a higher closeness level implies a shorter total distance from $v_{i}$ to all other nodes sequentially. In a supply network, closeness centrality can be considered a measure for flow speed (i.e., material or product) of a product distribution for an enterprise or as the relay speed of an entire supply chain.

#### 3.3.3 Betweenness centrality

Betweenness measures how often a node appears on the shortest paths between two other nodes in a network. Betweenness is introduced as a centrality measure to quantify such frequency as follows:

(5) $$C_{B}(v_{i})=\sum_{j=1,i\neq j}^{n}\sum_{k=1,i\neq k}^{j-1}\frac{\sigma_{jk}(v_{i})}{g_{jk}}$$

where $g_{jk}$ denotes the total number of shortest paths linking $v_{j}$ and $v_{k}$, and $\sigma_{jk}(v_{i})$ denotes the number of shortest paths that are involved in $v_{i}$. Betweenness centrality presents egocentric measures of a node between two other nodes in an entire network and presents sociocentric measures as it lies on the shortest available path. In supply networks, enterprises with high betweenness centrality play hub or pivotal roles that involve exchanging information with other relational points. However, high betweenness centrality resembles closeness, as the enterprises have the opposite function in a contractual supply network.

### 3.4 Support vector machine

A support vector machine (SVM) (Vapnik, 1995) belongs to the supervised learning theory group that is comparatively effective for classification, regression, and clustering tasks. Compared to other learning algorithms, an SVM can effectively manage high dimensional data space owing to its unique kernel ingredients. Different kernel functions can easily generate a set of decision functions even when the number of dimensions is greater than the total number of samples. During the data modeling phase, few data are learned in regards to the number of data points that are close to the data separating hyperplane, which we refer to as support vectors. Therefore, an SVM acts in the learning space as a memory efficient learning algorithm.

In this article, let us regard *n* as the i.i.d. sample: $\left(x_{c_{1}},x_{s_{1}},y_{1}),\cdots ,(x_{c_{n}},x_{s_{n}},y_{n}\right)$, where $x_{c_{i}}$ and $x_{s_{i}}$ denote customer and supplier features, respectively, and $y_{i}=\{+1,-1\}$ denotes the class label for $x_{c_{i}}$ and $x_{s_{i}}$. To obtain a better general decision surface, we first nonlinearly transform a set of input vectors $x=\{x_{c_{1}},x_{s_{1}},\cdots ,x_{c_{n}},x_{s_{n}}\}$ into a high-dimensional feature space, and the decision function *f* can be written as

(6) f(x)=h(x)+b

where $h(x)=\sum_{i=1}^{n}y_{i}\alpha_{i}(\phi (x_{i})\cdot \phi (x))$. Using the kernel trick, the inner product can be replaced with $K(x_{i}\cdot x)$. The final decision function in turn becomes:

(7) $$f(x)=\sum_{i=1}^{n}y_{i}\alpha_{i}K(x_{i}\cdot x)+b$$

where $K(x_{i}\cdot x)$, the kernel, is the most import ingredient of SVM theory. Among all of the hyperplanes, the best hyperplane (f(x)=0) can be found when the distance between two margin hyperplanes (f(x)=−1;f(x)=1) is maximized. In this study, an SVM is applied to extract customer–supplier relationships depending on the value of Eq. (7) in order to separate two classes as follows:

$$y_{i}=\left\{ \begin{array}{cc} 1\; & f(x_{i})\geq 1,\\ -1\; & f(x_{i})\leq -1. \end{array}\right.$$

When observed data are applied to an SVM, in order to find an appropriate kernel to map the observed data, several typical kernels of linear and nonlinear classification are proposed for the SVM as follows:

• Linear kernel: $K(x_{i}\cdot x_{j})=(x_{i}\cdot x_{j})$.
• Polynomial kernel: $K(x_{i}\cdot x_{j})={\left(x_{i}\cdot x_{j}+1\right)}^{d}$.
• Gaussian radial basis function: $K(x_{i}\cdot x_{j})=\exp{(-\gamma ∥x_{i}-x_{j})∥}^{2}$ where γ>0. This is sometimes parameterized using $\gamma =\frac{1}{2}\sigma^{2}$.

In contrast to prior researchers such as Mori et al. (2012) who only use the linear kernel, we also determine predictive accuracy levels by comparing with the polynomial kernel from hyperparameter d=2 after the increasing adjustment of the *d* value from 1 to d=5, and with the Gaussian radial basis function (RBF) from hyperparameter σ=0.2 after the increasing adjustment of the *σ* value from 0.2 to 1.0.

## 4 Experimental

### 4.1 Data preprocessing

#### 4.1.1 Data explanation

The experiments focus on supply chains in central Japan (data were provided by Tokyo Shoko Research Limited http://www.tsr-net.co.jp/en), one of the largest economic regions in the country. We select 182,538 enterprises including 598,721 transactions for central Japan, in which there are 10 prefectures and over 20 types of industrial categories.

Through the experiments, the main attributes are extracted from enterprise data, and their formats are described in Table 1. This is a full-scale data set for the entire Japanese industry that includes basic capital, founding date, employee quantity, location, industry category, sales, and profit data. These features are shown in the “Enterprise Attributes” rows. In addition to fundamental enterprise attributes, for each given enterprise, the data set also provides a list of customers (suppliers) numbering in the tens of thousands. Using these enterprise relationship data, we design features of customer–supplier relationships as shown in the “Network Centralities” rows in Table 1, which are described in Section 3.3. Although the degree measure is an important centrality, we exclude this measure from the explanatory variables, as we use it to separate the enterprises into small and medium enterprises (SMEs) and large enterprises (LEs) to evaluate the customer–supplier relationships found through our experimental testing (Section 4.3.3).

#### 4.1.2 Variable setup

In our experiment, we regard a pair of customer and supplier relationship as the response variable. As shown in Table 2, this is a Boolean variable where 1 denotes the existing transaction relation and −1 denotes the non-existing transaction relation.

We also design three types of explanatory variables, customer variables, supplier variables and dummy variables (Table 3). According to Section 3.4, let us consider $y=f(X_{c},X_{s},D_{cs})$ for an SVM model, where *y* denotes the customer–supplier relationship, $X_{c}$ and $X_{s}$ denote the variables shown in customer and supplier variable rows, respectively, and $D_{cs}$ denotes the dummy variables of common location and industry category, taking a value 0 or 1 to denote the degree of difference or similarity.

When only a real transaction relationship existing between customers and suppliers was observed, there were no negative samples in our data set. Therefore, we randomly generated the same number of customer and supplier pairs that present no customer–supplier relationship in the database to train the SVM model.

#### 4.1.3 SNA of supply network

**Figure 3:**
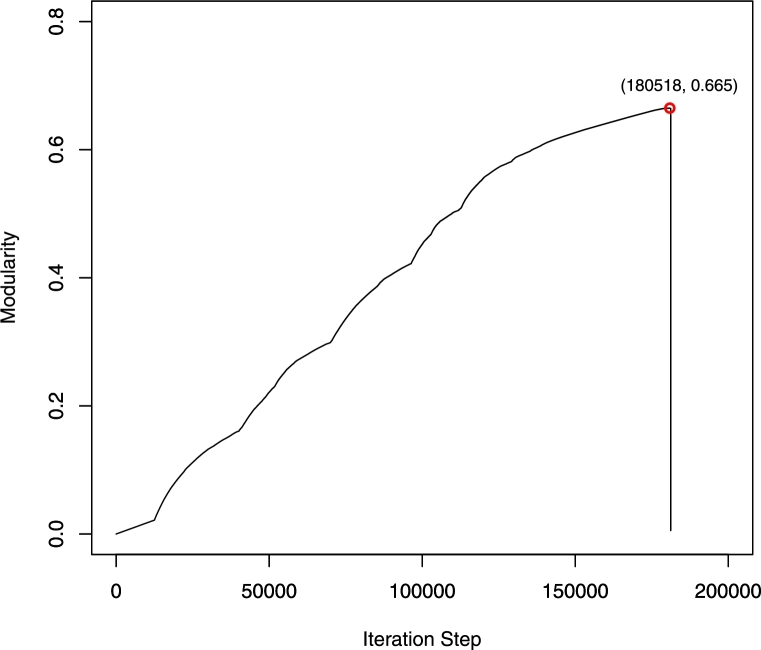
Identifying maximum modularity in a supply network using the Newman method.

**Figure 4:**
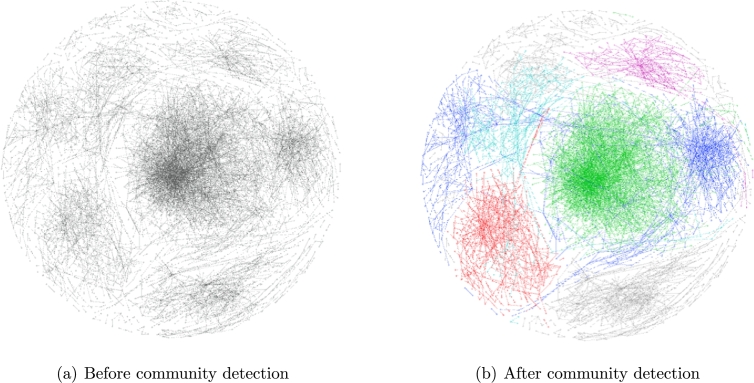
Supply network of the central area in the firework-like network chart.

This section investigates SNA as a technical approach to the analysis of customer–supplier relationships in supply chains by constructing supply community structures. We apply a fast modularity maximization algorithm (the Newman method) to detect and analyze communities in a supply network. This transaction structure is analyzed as described in Section 3.2, and the maximum modularity Q=0.665 can be found when the iterative step reaches 180,518 (Figure 3). According to this corresponding step, the supply network of central Japan is represented in a firework-like network chart (Figure 4 was created using the Gephi open source network analysis and visualization software program, https://gephi.github.io/), and five main communities are detected in this supply network by maximizing the modularity. Table 4 shows detailed results for each community, and communities M1 to M5 are highlighted in red, blue, green, light blue and purple as shown in Figure 4(b).

As a community differs from a general cluster depending on the number of nodes, a community is also dependent on the number of edges. We use an average clustering coefficient and average path length to represent a community, which provide a node overview and an edge overview, respectively. Although M3 does not include the most nodes because it includes the most edges, M3 has the maximum clustering coefficient and the shortest path length. In this experiment, we focus on community M3, which is more densely connected than other communities, and SVM is used to train an essential model for M3 before we use the Newman method to separate M3 into sparser sub-communities in Section 4.3.1.

In addition, using Eq. (3), Eq. (4) and Eq. (5), network centralities (degree, closeness and betweenness) are individually calculated for the enterprises in M3.

### 4.2 SVM model application

#### 4.2.1 Comparisons with different models

In this study, we use prior studies (Mori et al., 2012) as a benchmark whereby the linear kernel is employed to train an SVM classification model based on enterprise attributes. As denoted in Section 3.4, the polynomial kernel and Gaussian radial basis function (RBF) are also used in order to compare and find an appropriate kernel to map the observed data listed in this article. We test individual kernel tricks based on enumerative hyperparameters via 5-fold cross validation. According to the results listed in Table 5, the RBF kernel presents better predictive capacities than the others, and the highest accuracy level is obtained at σ=1.0. Here, the predictive accuracy level also depends on parameter *C*, which denotes the costs of constraint violations of rule trade-offs between the correct classification and model complexities. As we select C=1.0 as the default value for the experiments described in Table 5, we estimate parameter C=0.2 by increasing the value adjustment level from 0.2 to C=1.0 based on the RBF kernel with σ=1.0. The comparison results are shown in Table 6, and the highest accuracy level is obtained at C=1.0.

We also select an artificial neural network (ANN) as another benchmark. We estimate the *size* of units in the hidden layer from 2 to 10 and the parameter of weight *decay* from 0.2 to 2.0 through a grid search, and the optimal model can be obtained at *size* = 4 and *decay* = 2.0. The comparison results for ANN and SVM are shown in Table 7, and an SVM of σ=1.0 and C=1.0 is more accurate than an ANN of *size* = 4 and *decay* = 2.0. However, according to our experiments, the predictive accuracy level can be improved by using higher RBF kernel parameter values of *σ* and *C*. We stop at σ=1.0 and C=1.0 to follow the classification style of the SVM application in practice.

#### 4.2.2 Comparisons with different variable combinations

As the optimal SVM model is obtained when using an RBF kernel parameter of σ=1.0 and a cost of constraint violation of C=1.0, the following experiments are based on the same condition. This section introduces two more explanatory variables: closeness and betweenness. As described in Section 3.3, closeness is a measure that reflects an enterprise's ability to spread and relay information to others in a supply chain while accounting for the enterprise's sociocentric role, and betweenness measures an enterprise's ability to intervene or mediate interactions among other enterprises with respect to a supply chain and while accounting for both egocentric and sociocentric roles.

As shown in Table 8, we conduct a multivariate analysis to examine the association between enterprise attributes and network centralities. We then sequentially introduce groups of variables into the SVM as follows:

1. Variables of “Enterprise Attributes” only.
2. Variables of “Network Centralities” only.
3. Variables of “Enterprise Attributes” and “Network Centralities” together.

However, when NC variables are used alone, predictive accuracy levels are the lowest. After introducing NCs (closeness and betweenness) and the EA variables, the results (as shown in Table 8) improve significantly. The predictive accuracy level increases from 76.41% to 80.71%, and the predictive precision level increases from 74.71% to 77.94%. Additionally, in this case, the highest recall and F-value values of 85.65% and 81.61%, respectively, are achieved.

**Table 8:** Estimations of predicted customer–supplier relationships in M3.

	Enterprise Attributes (EA)	Network Centralities (NC)	EA & NC
Accuracy (%)	76.41	73.26	80.71
Positive (+)			
Precision (%)	74.71	78.92	77.94
Recall (%)	79.57	63.48	85.65
F-value (%)	77.06	70.36	81.61

### 4.3 Accuracy comparison and discussion

#### 4.3.1 Detection of sub-communities using the Newman method

**Figure 5:**
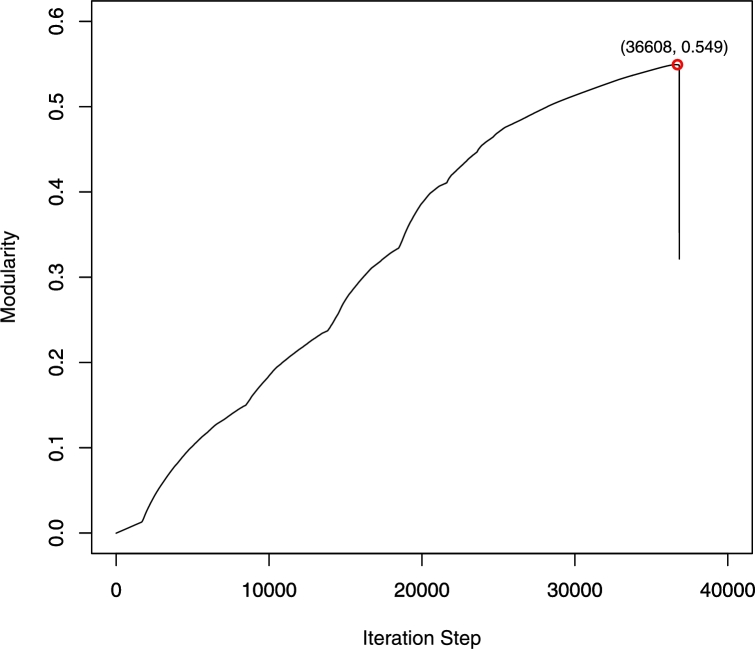
Identifying maximum modularity in M3 using the Newman method.

**Figure 6:**
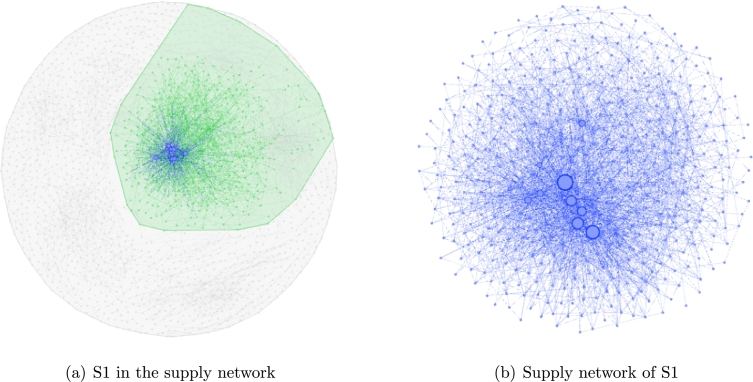
Supply network S1 in the firework-like network chart.

In this section, we apply the Newman method once more to separate M3 into sparser sub-communities. The maximum modularity level Q=0.549 can be found when the iterative step reaches 36,608 (Figure 5) and four sub-communities are detected from M3. Table 9 shows the division of each sub-community in detail. As shown in Figure 6, sub-community S1 is extracted from the main community M3 (Figure 6(a)), in which LEs (i.e. enterprises have the higher degree) are represented as large nodes (Figure 6(b)).

For each sub-community, network centralities (degree, closeness and betweenness) of individual enterprises are recalculated using Eq. (3), Eq. (4) and Eq. (5).

#### 4.3.2 Comparisons with different communities

This section compares the prediction performance of each sub-community shown in Table 4 and Table 9. For the same experimental testing conditions, we use an RBF kernel parameter of σ=1.0, a cost of C=1.0, and both EA and NC variables as explanatory variables. The numerical examples shown in Table 10 reveal the predictive performance of the original community and sub-communities in comparison with the accuracy values.

We first focus on results found for the original community and for its sub-communities (M1, M2, M3, M4 and M5). For each sub-community, the predictive results show lower accuracy levels than those of the original community when only EA variables are used, and higher accuracy values when only NC variables are used. However, the predictive accuracy derived when using EA & NC variables is also worse than that for the original community. Compared to the predictive accuracy rate of increase (EAs vs. EAs & NCs) which is improved by 4.08% (from 83.52% to 86.93%) for the original community, the rate of increase for each sub-community is improved by 6.96% (M1), 11.16% (M2), 5.63% (M3), 10.75% (M4) and 6.41% (M5). Next, we focus on the results for community M3 and for its sub-communities (S1, S2, S3 and S4), for which we draw the same conclusions. When introducing NC variables with EA variables, the predictive accuracy for each sub-community is improved by 7.18% (S1), 9.01% (S2), 8.15% (S3) and 9.39% (S4). All of these values are higher than that for community M3.

**Figure 7:**
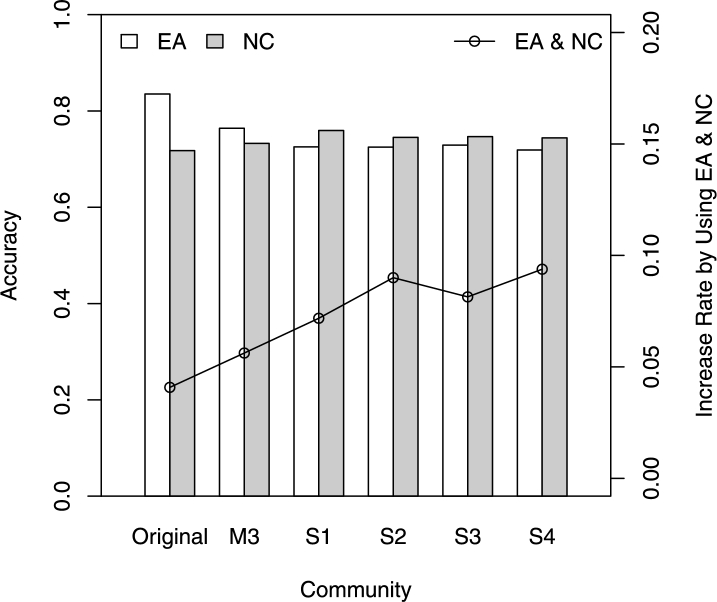
Comparisons of the predictive performance of the hierarchical communities.

The results are summarized and illustrated in Figure 7. The predictive accuracy based on the NC variables (gray histogram) and the rate of increase after introducing NC and EA variables (line) can be written as {S1, S2, S3, S4} > M3 > original community. On the opposite end, the predictive accuracy using EA variables (white histogram) can be written as {S1, S2, S3, S4} < M3 < original community. The Newman method offers an optimal community division that hierarchically separates an original community into sub-communities. Within sub-communities, nodes are connected much more densely than they are with other sub-communities, allowing the NC variables to play a leading role in predicting customer–supplier relationships. Therefore, when detecting sub-communities using Newman method, predictive performance levels can be dramatically improved (the rate of increase can be written as {S1, S2, S3, S4} > M3 > original community and can be represented as a polygonal line in Figure 7), as NCs of each enterprise in individual sub-communities are recalculated when fitting the new supply network.

#### 4.3.3 Comparisons between enterprises of varying sizes

In this section, we present our analyses of enterprises of different sizes from a network-based perspective, and sub-community S1 is used as the analysis target. Here, we employ the network centrality (degree) as the separating indicator rather than capital or employee quantity, as the capital and employee quantity are individual and attitudinal measures of enterprise size. A degree is defined as the number of edges from which a node connects to other nodes in a network, and it is recognized as an organizational and behavioral measure. An enterprise with a higher level of degree centrality has more direct relationships with other enterprises (customers or suppliers) in a supply network. Here, when an enterprise has more than 8 customers and suppliers (the average degree of S1 is 8.15), this enterprise is recognized as an LE. Otherwise, the enterprise is recognized as an SME. For a given customer–supplier relationship, enterprises are grouped by degree into 4 groups (LE–LE, LE–SME, SME–LE, SME–SME). Information on each group is shown in Table 11.

**Figure 8:**
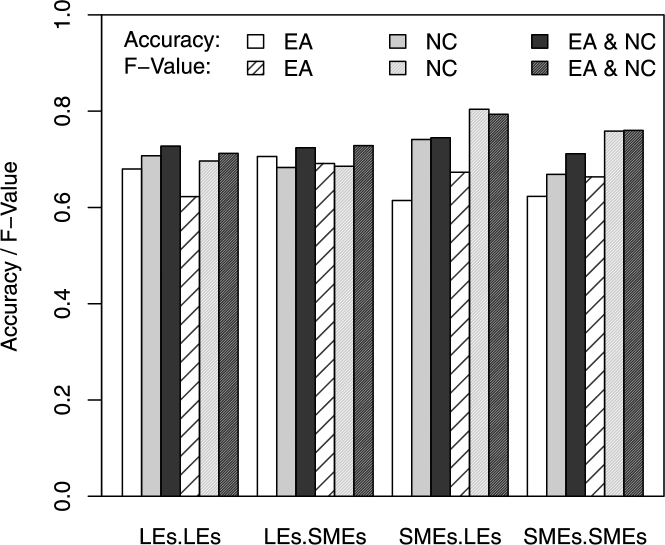
Comparisons of the predictive performance of the different enterprise groups.

In this experiment, we also compare accuracy, precision, recall and F-value measures for each group using the EA variables, NC variables and EA & NC variables, respectively (as shown in Table 12). The comparison results are presented in Figure 8 and are summarized as follows:

1. When only using EA variables: As the customers are LEs in LE–LE and LE–SME groups, the predictive accuracy (white histogram) is much higher than that for the SME–LE and SME–SME groups, for which customers are SMEs. The best and the worst accuracy levels are obtained for LE–SME and SME–LE groups, respectively. According to relationships like the customer–supplier relationship, the results show that when SMEs appear to be suppliers, it is easy for them to find customers as business partners when customers are LEs. By contrast, when SMEs appear to be customers, it is difficult for them to find suppliers as business partners, especially if SMEs wish to develop markets with LEs.
2. When only using NC variables: The predictive accuracy (gray histogram) and F-value (gray histogram with diagonal lines) for LE–LE and SME–LE groups and for SMEs are improved in comparison with those of case 1. However, for the LE–SME group, the predictive accuracy and F-value are not improved, and using NC variables alone can result in adequate predictive performance (almost the same as that for case 1).
3. When using EA & NC variables: The predictive accuracy (black histogram) of all the groups is improved relative to that of cases 1 and 2. Excluding the SME–LE group, the F-value (black histogram in diagonal lines) is also improved in the other groups relative to that of cases 1 and 2. While the F-value of case 3 for the SME–LE group is slightly worse than of case 2, it is still dramatically improved over case 1 by 17.90%.

These results present three operational improvements to the web system proposed by Mori et al. (2012), which automatically recommends a list of potential business partners for a given enterprise. First, we conduct an SNA to structurally analyze a supply network and to introduce network centrality to machine learning to predict customer–supplier relationships. Compared to the benchmark measure listed in Mori et al. (2012), when we applied closeness centrality and betweenness centrality values as new explanatory variables, the predictive accuracy was dramatically improved. Second, one issue remained unaddressed in Mori et al. (2012)'s study. When a user searches for a new enterprise with enterprise attributes that are not included in a database, web systems find no business partners. Here, we propose a learning model with network centralities of high predictive accuracy that uses network centralities as explanatory variables. In searching for a new enterprise based on its name, a web system can return a recommended list of business partners, by identifying enterprise actors of a certain business community bases on network centralities. Third, unlike LEs, for SMEs, it is much more difficult to find potential business partners and to develop new business opportunities using enterprise attributes alone. Our proposed method offers a higher degree of predictive accuracy in terms of SME–LE and SME–SME relationships than the original model, and in turn, SMEs may obtain effective recommendations from web systems that can lead them to future enterprise success.

## 5 Conclusions

This article proposes an SNA-based prediction method for identifying business partners. We examined several important methodological issues related to SNA as an alternative means of analyzing supply chains relative to the traditional linear perspective, and we explored a machine learning approach to the supply networks based on customer–supplier relationships. First, we found that the SNA approach not only offers a new perspective on customer–supplier relationships as network structures, and also allows NCs to reveal and consider dynamic features of individual enterprises. In contrast to prior studies that have used EAs alone as explanatory variables, our proposed approach, which combines NCs and EAs, sufficiently predicts customer–supplier relationships. Second, we demonstrated the effectiveness of our proposed approach when applied to LEs and SMEs. From our experiments, we found that the integration of NCs with EAs can improve levels of predictive performance for all combinations of customer–supplier relationships. Especially for SMEs which are vulnerable groups in business environments, the predictive results are accurate enough for SMEs to develop partnerships with LEs and other SMEs. We believe that these findings can familiarize other researchers with NCs to stimulate new approach on the design of supply chains and to also provide insight into the further development of business partner recommendation systems based on machine learning.

Our aim for the future extension of this article is to achieve the levels of highest accuracy levels possible. As a limitation of this article, while our proposed method outperforms other methods, its predictive performance must still be estimated in search of the optimal combination of parameters. We also plan to generate new variables (i.e., text information on enterprises that can be extracted from company websites and news media sources) and to then customize mapping approaches for these variables to gain perspective on actual business conditions.

## Declarations

### Author contribution statement

Yi Zuo: Conceived and designed the experiments; Performed the experiments; Analyzed and interpreted the data; Contributed reagents, materials, analysis tools or data; Wrote the paper.

Junichiro Mori and Yuya Kajikawa: Conceived and designed the experiments; Analyzed and interpreted the data; Contributed reagents, materials, analysis tools or data.

### Competing interest statement

The authors declare no conflict of interest.

### Funding statement

This research was conducted with the support by RIETI (Research Institute of Economy, Trade, and Industry), and also by COI STREAM, Ministry of Education, Culture, Sports, Science, and Technology. This research was also partially supported by JSPS KAKENHI Grant Number JP26330344.

### Additional information

No additional information is available for this paper.

## Figures and Tables

**Table 1:** Definitions and explanations of the variables.

Type	Feature	Format
Enterprise Attributes	Capital	100k – 397,049,999k (JPY)
	Founding Date	1906 – 2012 (YYYY)
	Number of Employees	1 – 69,125 (#)
	Location Code	40 – 50 (ten prefectures)
	Industry Category Code	0100 – 9999
	Sales	0k – 8,241,176,000k (JPY)
	Profit	−5,351,000k – 79,164,000k (JPY)
Network Centralities	Degree	Eq. (3)
	Closeness	Eq. (4)
	Betweenness	Eq. (5)

**Table 2:** Response variable.

Customer–Supplier Relationship	{−1,1}

**Table 3:** Explanatory variables.

Customer Variables	Capital, Founding Date,
	Number of Employees,
	Sales, Profit,
	Closeness, Betweenness
Supplier Variables	Capital, Founding Date,
	Number of Employees,
	Sales, Profit,
	Closeness, Betweenness
Dummy Variables	Common Location Code,
	Common Industry Category Code

**Table 4:** Detection results for each community in central Japan.

Community No.	Nodes (#)	Edges (#)	Avg. Clustering Coefficient	Avg. Path Length
M1	41,594	123,955	0.032	8.325
M2	40,291	113,546	0.022	8.843
M3	36,832	137,301	0.041	5.691
M4	22,818	56,443	0.023	9.434
M5	20,469	61,799	0.035	7.913

**Table 5:** SVM performance with the linear / polynomial / RBF kernel trick.

Kernel Type	Linear	Polynomial (*d*)				RBF (*σ*)				
Parameter	–	2	3	4	5	0.2	0.4	0.6	0.8	1.0
Accuracy (%)	74.00	73.83	74.99	75.03	74.26	75.23	75.62	76.04	76.24	76.41

**Table 6:** SVM performance based on different costs of constraint violation.

Kernel Type	RBF (σ=1.0)				
Parameter *C*	*C* = 0.2	*C* = 0.4	*C* = 0.6	*C* = 0.8	*C* = 1.0
Accuracy (%)	75.51	75.95	76.10	76.23	76.41

**Table 7:** Performance comparisons of between ANN and SVM.

Model	ANN (*size* = 4, *decay* = 2.0)	SVM (*σ* = 1.0, *C* = 1.0)
Accuracy (%)	70.95	76.41

**Table 9:** Detection results for each community in M3.

Community No.	Nodes (#)	Edges (#)	Avg. Clustering Coefficient	Avg. Path Length
S1	9,830	40,038	0.061	4.982
S2	7,713	18,767	0.037	7.416
S3	6,717	19,179	0.045	6.356
S4	6,661	14,582	0.036	8.778

**Table 10:** Estimations of predictive performance for different communities.

Community	Accuracy (%)		
EA	NC	EA & NC
Original	83.52	71.76	86.93

M1	80.73	74.17	86.35
M2	75.43	74.54	83.85
M3	76.41	73.26	80.71
M4	70.70	74.94	78.30
M5	80.85	73.53	86.03

S1	72.55	75.94	77.76
S2	72.50	74.52	79.03
S3	72.91	74.66	78.85
S4	71.90	74.42	78.65

**Table 11:** Degree-specific results for each group.

Customer–Supplier Relationship, Degree-specific	Nodes (#)	Edges (#)
LE–LE Group	2,317	16,080
LE–SME Group	8,007	12,973
SME–LE Group	6,106	7,672
SME–SME Group	4,004	3,313

**Table 12:** Comparisons between enterprises by degree based on EA, NC and EA & NC variables.

Customer–Supplier Relationship		EA	NC	EA & NC
LE–LE	Accuracy (%)	67.98	70.73	72.73
Positive (+)			
Precision (%)	82.79	77.23	80.65
Recall (%)	49.88	63.41	63.79
F-value (%)	62.25	69.64	71.24

LE–SME	Accuracy (%)	70.59	68.31	72.39
Positive (+)			
Precision (%)	79.67	73.88	79.85
Recall (%)	61.18	63.97	65.40
F-value (%)	69.12	68.57	72.85

SME–LE	Accuracy (%)	61.44	74.11	74.47
Positive (+)			
Precision (%)	62.18	68.10	70.57
Recall (%)	73.34	98.11	90.62
F-value (%)	67.30	80.40	79.35

SME–SME	Accuracy (%)	62.30	66.87	71.15
Positive (+)			
Precision (%)	62.84	61.70	67.82
Recall (%)	70.28	98.46	86.48
F-value (%)	66.35	75.86	76.02
